# The Korean hip fracture registry study

**DOI:** 10.1186/s12891-023-06546-z

**Published:** 2023-06-02

**Authors:** Jung-Wee Park, Yong-Chan Ha, Jin-Woo Kim, Tae-Young Kim, Ji Wan Kim, Seung-Hoon Baek, Young-Kyun Lee, Kyung-Hoi Koo

**Affiliations:** 1 Department of Orthopaedic Surgery, Seoul National University Bundang Hospital, Seongnam, South Korea; 2 Department of Orthopaedic Surgery, Seoul Bumin Hospital, Seoul, South Korea; 3 Department of Orthopaedic Surgery, Nowon Eulji Medical Center, Seoul, South Korea; 4 Department of Orthopaedic Surgery, Konkuk University School of Medicine, Seoul, South Korea; 5 Department of Orthopaedic Surgery, Asan Medical Center, University of Ulsan College of Medicine, Seoul, Korea; 6 Department of Orthopedic Surgery, School of Medicine, Kyungpook National University, Kyungpook National University Hospital, Daegu, South Korea

**Keywords:** Registry study, Hip fracture, Osteoporotic fracture, Fracture liaison service, Second fracture

## Abstract

**Background:**

The purpose of the Korean Hip Fracture Registry (KHFR) Study is to establish a nationwide, hospital-based prospective cohort study of adults with hip fracture to explore the incidence and risk factors of second osteoporotic fractures for a Fracture Liaison Service (FLS) model.

**Methods:**

The KHFR, a prospective multicenter longitudinal study, was launched in 2014. Sixteen centers recruited participants who were treated for hip fracture. The inclusion criteria were patients, who were treated for proximal femur fracture due to low-energy trauma and aged 50 or more at the time of injury. Until 2018, 5,841 patients were enrolled in this study. Follow-up surveys were conducted annually to determine occurrence of second osteoporotic fracture, and 4,803 participants completed at least one follow-up survey.

**Discussion:**

KHFR is a unique resource of individual level on osteoporotic hip fracture with radiological, medical, and laboratory information including DXA (dual energy x-ray absorptiometry), bone turnover marker, body composition, and hand grip strength for future analyses for FLS model. Modifiable factors for mortality after hip surgery is planned to be identified with nutritional assessment and multi-disciplinary interventions from hospitalization to follow-ups. The proportions of femoral neck, intertrochanteric, and subtrochanteric fractures were 517 (42.0%), 730 (53.6%), and 60 (4.4%), respectively, from 2014 to 2016, which was similar in other studies. Radiologic definition of atypical subtrochanteric fracture was adopted and 17 (1.2%) fractures among 1,361 proximal femoral fractures were identified. Internal fixation showed higher reoperation rate compared to arthroplasty in unstable intertrochanteric fractures (6.1% vs. 2.4%, p = 0.046) with no significant difference in mortality. The KHFR plans to identify outcomes and risk factors associated with second fracture by conducting a 10-year cohort study, with a follow-up every year, using 5,841 baseline participants.

**Trial registration:**

Present study was registered on Internet-based Clinical Research and Trial management system (iCReaT) as multicenter prospective observational cohort study (Project number: C160022, Date of registration: 22th, Apr, 2016).

## Key messages

The KHFR study, which is a representative of the Korean hip fracture patients, showed that basic epidemiologic information of hip fracture including atypical hip fracture in Korea.

Perioperative nutritional assessment and management by multi-disciplinary interventions could decrease malnutrition and mortality risk.

Internal fixation in unstable intertrochanteric fracture was associated with higher reoperation rate, comparing with bipolar hemiarthroplasty, while mortality after surgery was not significantly differ according to the type of surgery.

Prospective cohort and representative registry should be established to develop effective secondary prevention program such as Fracture Liaison Service.

## Background

A hip fracture is one of the major osteoporotic fractures and the leading cause of disability for elderly population worldwide [1–3]. Treating osteoporosis and preventing fractures are therefore crucial in maintaining quality of life [4] and reducing medical costs associated with fracture treatment and disability in the elderly [1, 5]. A previous fracture is known to be the most important and strongest risk factor of osteoporotic fracture [6]. Secondary prevention, which is the prevention of subsequent fracture in patients with the previous fracture, is imperative [7–16]. The need for effective secondary prevention and appropriate management of previous fracture has grown rapidly [17].

The risk of hip fracture in Korean population has been considered as moderate with reference to global data [18], and hip fracture event should be treated with secondary prevention and osteoporosis treatment [8–16]. Fracture Liaison Service (FLS), a coordinator-based multidisciplinary management, has internationally been highlighted as one of the representative efforts for secondary prevention [8, 9, 12–14]. The registry of patients with osteoporotic fractures is essential to establish and maintain the system for secondary prevention [8, 9, 12–14].

In the early 1990s, several cohort studies including patients with osteoporosis or hip fracture began in Korea, [19–21] but were localized to small areas and were not sufficiently large enough to determine scale of subsequent fracture. Moreover, the studies did not focus on secondary prevention after hip fracture, which is the most dangerous osteoporotic fracture [19–21].

The Korean Hip Fracture Registry (KHFR) Study Group was established in 2014 with financial support from the Korea Health Technology R&D Project through the Korea Health Industry Development Institute (KHIDI), funded by the Ministry of Health & Welfare, Republic of Korea. The Study Group conducted a large-scale cohort study of Korean patients who were aged 50 years or more and were treated for proximal femur fracture from 16 academic tertiary hospital throughout Korea, with the following goals: (1) to evaluate outcome of mobility and mortality after fracture surgery (2) to establish a registry for patients with proximal femur fracture, (3) to evaluate scale of secondary hip fracture after prior hip fracture, (4) to evaluate the occurrence of atypical femoral fracture, one of the possible adverse events of osteoporosis treatment and (5) to form the basis for a prospective cohort study to explore the incident second osteoporotic fractures for a FLS model.

## Methods

### Cohort description

**Fig. 1:**
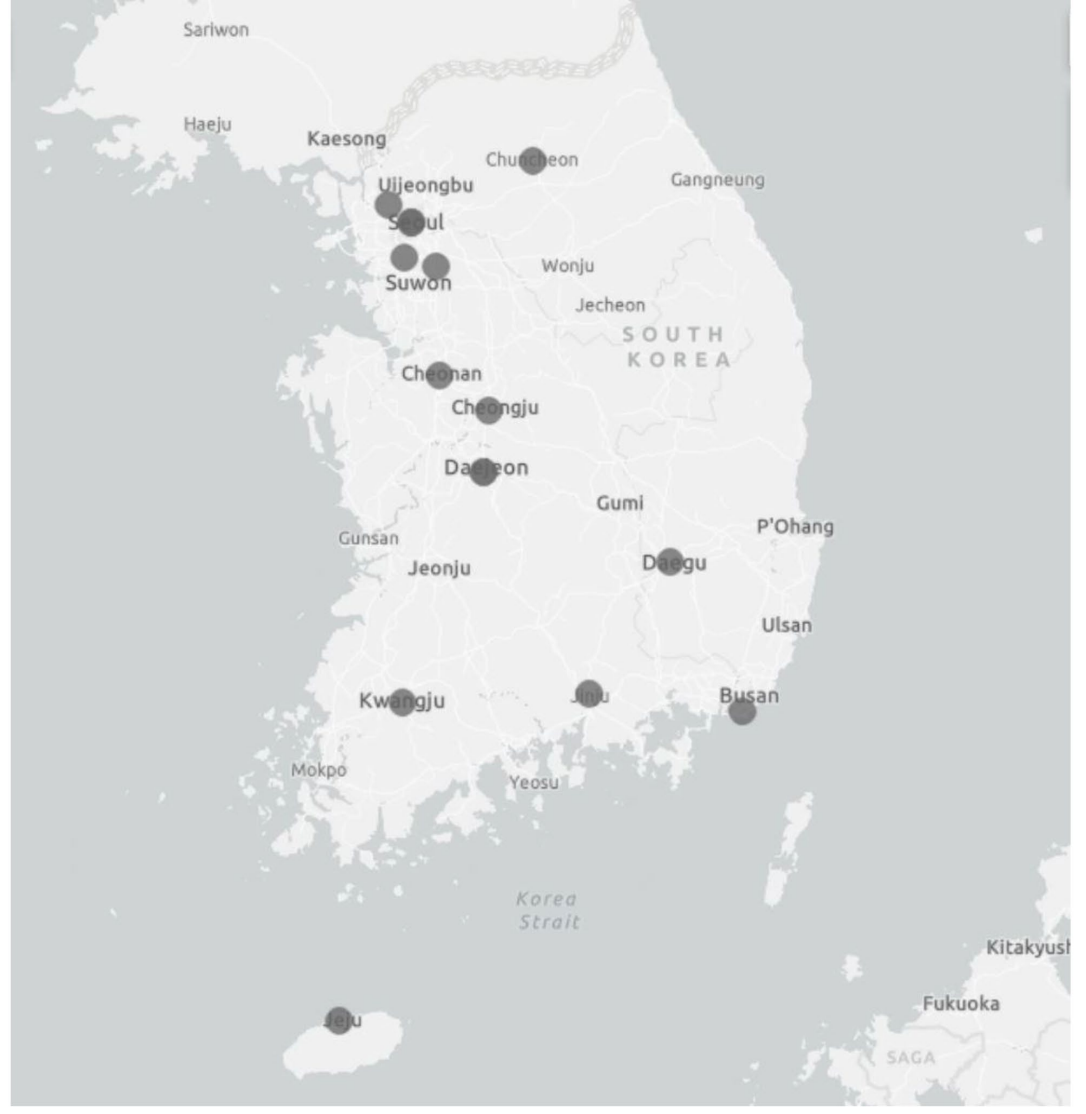
Study areas for the Korean Hip Fracture Registry study

Sixteen tertiary hospitals representing each area of South Korea have participated in this KHFR study. The study was designed as a prospective, multicenter, hospital-based, observational cohort study between July 2014 and 2018 (Fig. 1). The patients who were treated for proximal femur fracture due to low-energy trauma and aged 50 years or more at the time of injury were included. Younger patients or those fractured from high-energy trauma differ from osteoporotic hip fractures in nature and were therefore excluded in this investigation.

Of the 5,841 participants, 532 (9.1%) joined the study in 2014, 1,149 (19.7%) joined in 2015. 1,233 (21.1%) joined in 2016, 1,734 (29.7%) joined in 2017, and 1,193 (20.4%) joined in 2018. The mean age was 78.3 (50–104) years, and 4,255 participants (72.8%) were female.

Surgeons decided on the type of surgery (internal fixation and hip arthroplasty) to be performed based on the stability of the fracture type, patient’s age, activity level before the injury, osteoporosis and underlying comorbidities.

After hip fracture surgery, patients were encouraged to walk using assistive devices; walker or crutches from the second postoperative day. The assistive devices were recommended to be used for one month.

Osteoporosis was treated with medication by physicians as part of routine clinical practice, according to their clinical judgement and national reimbursement criteria.

This study was performed in accordance with The Code of Ethics of the World Medical Association (Declaration of Helsinki). The study protocol was approved by the Ethics Committee of each hospital.

All patients visited the corresponding institutions for head-on interviews, physical examinations and laboratory tests for the baseline survey. At the postoperative follow-ups, participants primarily visited their centers, but we also conducted home visits, telephone checks, and proxy interviews (in this order) if the visit to the institution was not feasible.

All patients were informed of the results of their laboratory tests and radiologic exams including plain radiographs and dual energy X-ray absorptiometry (DXA).

**Fig. 2:**
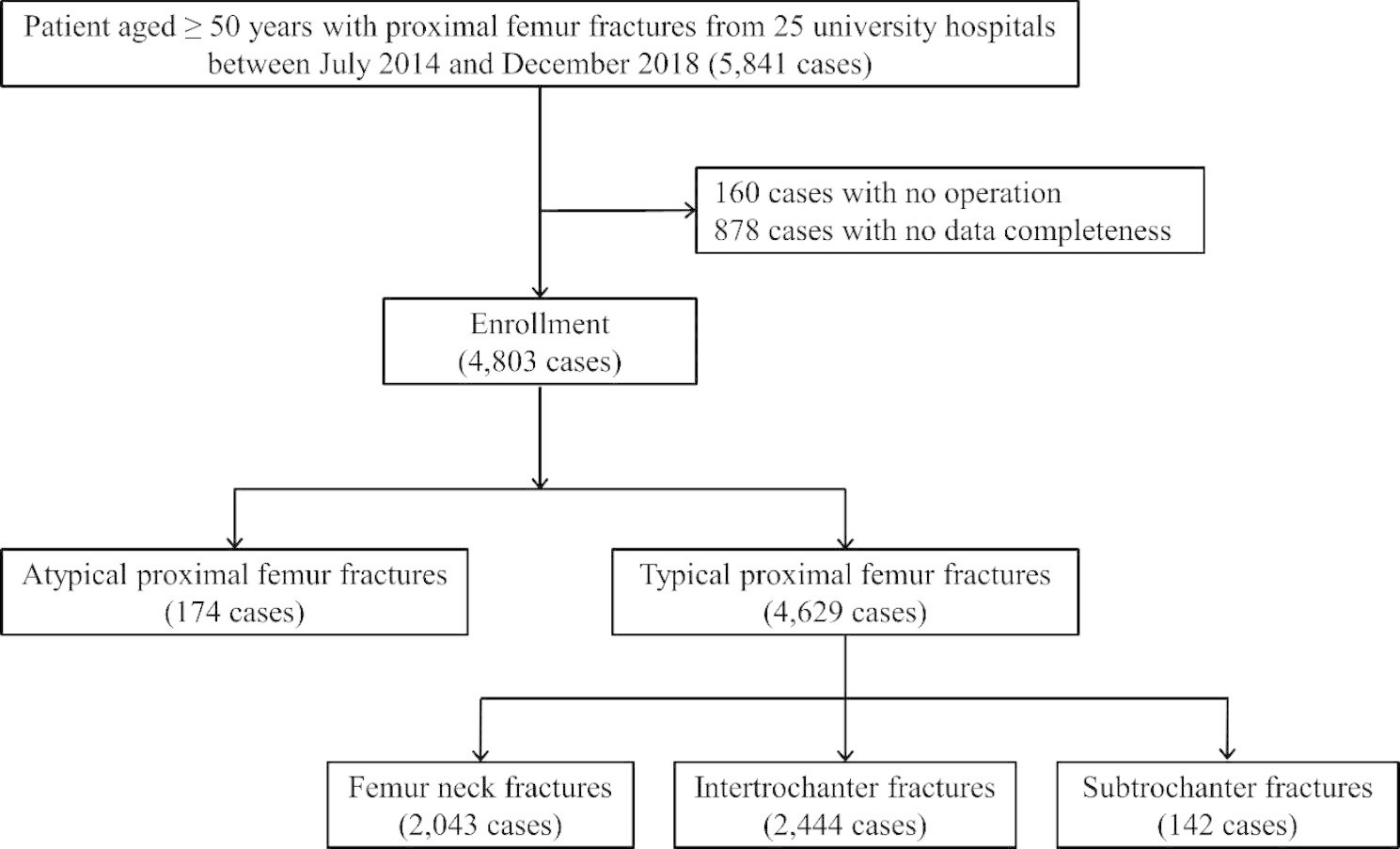
Flowchart of the Korean Hip Fracture Registry study

We conducted annual follow-up surveys after surgery with the same protocols for clinical and radiological evaluations at baseline, as well as same questionnaire. Among 5,841 patients enrolled between 2014 and 2018, 4,803 participants completed at least one-year follow-up survey. (Fig. 2) Currently, the KHFR study plans to register subjects and maintain regular annual follow-up.

### Data collection and variables

Categories for measurements conducted at baseline and follow-up evaluations are listed in Table 1.

**Table 1:** Measurement in the Korean Hip fracture Registry Cohort Study

Basic demographic information	Age, gender, date of birth, height, weight, BMI* Date of injury Injury mechanism Location at injury Concomitant injury Previous history of fracture
Radiologic evaluation	Injured side Diagnosis Type of fracture Feature of fracture (typical vs. atypical) Spine morphology
Past medical history	Neurologic disorder Cardiovascular disease Endocrine disease Respiratory disease Renal disease Ophthalmic disease Spine disease Hepatobiliary disease Hematologic disease Connective tissue disease
Family status	Residency (rural or urban) Type of housemate
Previous mobility	Koval classification
Previous activity/ independence	Functional independence measure scale
ASA* score	Class 1 ~ 4
Quality of life	EQ-5D
Evaluation of bone metabolism	Bone mineral density using DXA* Bone turnover marker (cTx*, nTx*, osteocalcin, bone specific ALP*)
Evaluation of vertebral fracture	Spine x-ray
Laboratory	25(OH) vitamin D PTH* Total calcium Phosphorus Albumin Fasting plasma glucose Total cholesterol HDL* cholesterol

BMI; Body mass index, ASA; American Society of Anesthesiologists, DXA; Dual Energy X-ray Absorptiometry cTx; C-telopeptide, nTx*; N-telopeptide, ALP*; Alkaline phosphatase, PTH*; Parathyroid hormone, HDL*; High density lipoprotein

The KHFR baseline study included radiologic features of fracture as well as baseline demographic information. It covered a wide range of bone health-related parameters such as bone mineral density (BMD) measurements, radiologic vertebral fracture and biochemical markers of bone turnover.

Serum analysis including complete blood count, electrolyte, admission panel, and bone turnover markers was performed in all patients. Biochemical bone turnover markers (C-telopeptide, N-telopeptide, osteocalcin, and bone specific alkaline phosphatase (ALP)) and other more specific markers such as 25(OH) vitamin D and PTH were evaluated. Serum analysis was initially performed at the time of surgery. Afterwards, it was performed at the annual visit with the DXA scan.

Follow-up surveys were designed to detect outcomes such as mobility/activity change, BMD, incidence of subsequent osteoporotic fracture (hip, vertebral, wrist, and proximal humerus fractures).

In terms of osteoporosis treatment, type of medication, route of administration, dose, interval, and duration of use were recorded.

The patients’ data were collected at the central office using the Korea National Institute of Health web-based system (Internet-based Clinical Research and Trial management system (iCReaT), Cheongju, Korea).

For the patients who are unable to communicate or have cognitive disorders, the descriptive data was acquired by interviewing the family of the patients and searching medical records.

### Assessment of outcomes

Surgical parameters including operation time, estimated blood loss, and amount of transfusion were recorded. Surgical complications including intraoperative problems, nonunion, infection, delirium, venous thromboembolism (VTE), and reoperation were evaluated.

Subject’s mobility and independence according to the Koval’s Classification [22] and Functional independence measure scale [23] were obtained through interviews at each follow-up. The ambulatory levels were categorized into outdoor ambulators (Koval’s grade 1,2,3) and housebound patients (Koval’s grade 4,5,6).

The time of a fracture event, the site of fracture, and the situation in which the fracture occurred were also acquired with interviews at each follow-up survey. Osteoporotic fracture was defined as a fracture that occurred without strong external force or was caused by falling from height level [3] and was diagnosed by a physician with radiographic examination. A vertebral fracture was also evaluated radiologically at baseline and each of the follow-up evaluation.

BMD of lumbar vertebrae and the proximal femur were obtained annually by using DXA (Hologic or Lunar GE) by physicians as part of routine clinical practice.

Serum osteocalcin (OC) and bone-specific ALP were measured as markers of bone formation, and serum type I collagen C-terminal telopeptide (CTX), serum type I collagen N-terminal telopeptide (NTX), as markers of bone resorption. Korean government has reimbursed one of each kind of bone marker (one of bone formation markers and the other of bone resorption markers).

Osteoporosis was treated with medication as part of routine clinical practice, according to the clinical judgement and national reimbursement criteria. Information on the type of medication, dose, interval, duration of medication was evaluated in each survey.

Patients or their family, who were unable to return for a follow-up evaluation, were contacted with a telephone questionnaire survey. When contacting via telephone, the death of the cohort patient was also inquired.

## Discussion

### Modifiable factors for mortality after hip fracture surgery

Hip fracture is known to be associated with high mortality and functional disability [1, 4, 24, 25]. Several demographic factors including male, older age, low BMI, cognitive impairment, delayed surgery, severe comorbidity, and poor preinjury mobility have been known to be associated with higher mortality in operated hip fracture patients [24, 26–29]. To lower the mortality after hip fracture surgery, the modifiable risk factors should be identified. From our prospective multicenter cohort study, nutritional assessment and management by multi-disciplinary interventions from hospitalization to follow- ups could decrease malnutrition and mortality risk [30].

### Proportion of femoral neck, intertrochanteric and subtrochanteric fracture

The proximal femur has a complex anatomy, consisting of the femoral neck, intertrochanteric, and subtrochanteric areas [31]. Several epidemiological studies showed that proportions of each fracture were constant, regardless of countries [32–35]. Among 1,361 proximal femoral fractures registered between 2014 and 2016, 571 femoral neck fractures (42.0%), 730 intertrochanteric fractures (53.6%), and 60 subtrochanteric fractures (4.4%) were observed [36]. These proportions were similar with those of the previous study using Korean national claim database, [31] and comparable with those of other countries [32–35].

### Scale of occurrence of atypical femur fracture

Atypical femur fracture (AFF) was defined as a transverse or short oblique, non-comminuted subtrochanteric/diaphyseal fracture in an area of locally thickened cortices with unicortical beak in radiographs, according to the definition of ASBMR taskforce [37].

Several epidemiologic studies showed that the incidence rate of AFF varies from 76 to 310 per 100,000 person-years [38–42]. However, they used international classification of disease (ICD) code system to identify atypical subtrochanteric fractures(ASF), instead of radiologic definition of ASBMR. To determine the exact scale of occurrence of ASF, a radiologic review is essential. We evaluated the occurrence of ASF in South Korea, by using radiologic definition of ASF [43]. Among 1361 patients with proximal femoral fractures due to low-energy trauma, 17 fractures (1.2%) were identified as ASF. Higher BMI and use of bisphosphonate before injury were associated factor with occurrence of ASF [43]. The occurrence of ASF was rare, and its scale could be comparable with those of Western countries [44–48].

### Treatment of unstable intertrochanteric fracture

The treatment of unstable intertrochanteric fractures in the elderly is challenging and technically demanding due to old age, underlying comorbidities, osteoporosis, and insufficient bony support at calcar [49–55]. Furthermore, the ideal treatment for intertrochanteric fractures in elderly osteoporotic patients remains controversial [49–55].

The benefits of internal fixation or joint preservation procedure, continue to be debated because of loss of fixation related to calcar defect, lateral wall involvement and severe osteoporosis, which often eventually require conversion to hip arthroplasty [53–56]. Although hip arthroplasty has theoretical advantages, longer operation time, higher mortality and late dislocation remain problematic. Internal fixation and hip arthroplasty have their pros and cons and these should be considered by clinicians when deciding treatment. Well-designed randomized controlled trials should provide the highest level of evidence regarding the merits of procedures, but they are not always possible especially in elderly patients because of little clinical reliability. Our hospital-based multicenter prospective cohort study compared outcomes between internal fixation and hip arthroplasty in patients with unstable intertrochanteric fractures in the elderly aged ≥ 65 years. After investigating 571 unstable intertrochanteric fractures, among the registered 1,047 patients between July 2014 and June 2016, our results showed that reoperation rate in the internal fixation was higher than that in the bipolar hemiarthroplasty group (6.1% vs. 2.4%, *p* = 0.046), while mortality after surgery was not significantly different according to the type of surgery [57].

Strengths of the KHFR study are the large sample size and representative institutes covering entire South Korea to reduce the effects of regional differences in fracture occurrence. This study used the most accurate diagnostic criteria for osteoporotic hip fracture proven by radiographs and medical records, especially for atypical femoral fracture and vertebral fracture. In addition, this prospective cohort will be the first cohort registry for secondary prevention for patients with osteoporotic hip fracture in East Asia. This could provide fundamental data for future study on secondary prevention and FLS model from East Asia.

KHFR study has limitations. The enrolled subjects were not randomly selected from the Korean general population, although the voluntarily participating institutes are distributed throughout Korea. In addition, only elderly osteoporotic hip fracture patients were included in this study. The younger patients with fracture caused by high-energy trauma consist a different cohort with difference in demographics and treatment principles.

KHFR study is the only cohort study representative of the Korean population with osteoporotic hip fracture and provides basic information for secondary prevention after first hip fracture, so-called FLS model in Korea. This could be the first audit registry for FLS program in East Asia. This cohort also included representative biochemical markers of bone metabolism and wide range and depth of individual-level clinical information for future studies.

## Data Availability

Currently, the KHFR dataset can be accessed through the Internet-based Clinical Research and Trial management system (iCReaT) of the Korea National Institute of Health website [https://icreat.nih.go.kr/icreat/webapps/com/hismainweb/jsp/cdc_n2.live?indexYN=Y&langtp=K]. The KHFR database is freely available and encourages new collaborations after approval of KHFR committee. Potential collaborators are invited to contact YCH at the administrative office of the KHFR Study Group at the Department of Orthopedic surgery, Seoul Bumin Hospital, Seoul, Korea.

## Abbreviations

KHFR: Korean Hip Fracture Registry
FLS: Fracture Liaison Service
DXA: Dual energy X-ray absorptiometry
AFF: atypical femoral fracture
KHIDI: Korea Health Industry Development Institute
BMD: bone mineral density
ALP: alkaline phosphatase
iCReaT: Internet-based Clinical Research and Trial management system
VTE: venous thromboembolism
OC: osteocalcin
CTX: C-terminal telopeptide
NTX: N-terminal telopeptide
BMI: body mass index
ASF: atypical subtrochanteric fracture
